# Healthy Nordic diet and risk of disease death among men: the Kuopio Ischaemic Heart Disease Risk Factor Study

**DOI:** 10.1007/s00394-020-02188-2

**Published:** 2020-02-03

**Authors:** Hanna-Mari Tertsunen, Sari Hantunen, Tomi-Pekka Tuomainen, Jyrki K. Virtanen

**Affiliations:** Institute of Public Health and Clinical Nutrition, University of Eastern Finland, P.O. Box 1627, 70211 Kuopio, Finland

**Keywords:** Baltic Sea Diet Score, Nordic diet, Cancer, Cardiovascular disease, Mortality, Population study, Prospective study

## Abstract

**Purpose:**

To investigate the association between healthy Nordic diet and risk of disease death in middle-aged and older men from eastern Finland.

**Methods:**

A total of 1547 men aged 42–60 years and free of cardiovascular disease (CVD), cancer and type 2 diabetes at baseline in 1984–1989 were included. Diet was assessed with 4-day food records at baseline and the healthy Nordic diet score was calculated based on the Baltic Sea Diet Score. The incidence of death was assessed by a computer linkage to the national cause of death register. Cox proportional hazards regression analyses were used to estimate the associations between the healthy Nordic diet score and mortality.

**Results:**

During the mean follow-up of 23.6 years (SD 7.0), 576 men died due to disease: 250 due to CVD, 194 due to cancer and 132 due to other diseases. The multivariable-adjusted hazard ratios (95% confidence interval) in the lowest vs. the highest quartile of the healthy Nordic diet score were 1.27 (1.01–1.59) for any disease death (*P*-trend across quartiles < 0.001), 1.39 (0.99–1.97, *P*-trend = 0.049) for CVD death, 1.26 (0.84–1.89, *P*-trend = 0.316) for cancer death and 1.04 (0.65–1.68, *P*-trend = 0.563) for other disease deaths.

**Conclusions:**

In this prospective population-based cohort study among middle-aged and older men, low adherence to a healthy Nordic diet was associated with a higher risk of any disease death, possibly largely attributable to higher CVD mortality.

## Introduction

Due to cultural and geographical differences, the composition of diet varies in different populations and thus associations between single nutritional factors and health outcomes are challenging to estimate. Dietary scores have been developed to consider the cumulative effects and interactions between several food items and nutrients. They represent a summary value of consumed foods and nutrients and characterize a measure of adherence to a predefined diet [1].

The Mediterranean-style diet has been most frequently used to describe a healthy diet [2]. The Mediterranean Diet Score is based on the traditional diet used in the Mediterranean countries and is characterized, for example, by high intake of olive oil [3]. However, due to differences in food cultures and local resources, the Mediterranean diet may not be easily adapted to other geographical regions. The Baltic Sea Diet Score is one of the diet scores that was developed to characterize a diet based on typical Nordic foods consumed in Finland. A high Baltic Sea Diet Score is characterized by high consumption of berries and fruits, whole grains, vegetables, rapeseed oil, fish, low-fat dairy and low consumption of processed meat and alcohol [4]. Earlier studies have shown that a higher Baltic Sea Diet Score is associated with, for example, lower risk of abdominal obesity [5], better physical capacity in old age [6] and lower risk of elevated C-reactive protein concentration [7]. Other similar diet scores that have been developed to define a healthy Nordic diet have also observed beneficial associations with disease risk factors [8–10], although less is known of the associations with risk of diseases or mortality and the findings are inconclusive [11–19].

Therefore, the aim of this study was to examine the association between a healthy Nordic diet and risk of death due to cardiovascular diseases (CVD), cancer, and other diseases in the Kuopio Ischaemic Heart Disease Risk Factor Study (KIHD), a population of middle-aged and older men from eastern Finland.

## Materials and methods

### Study population

**Fig. 1:**
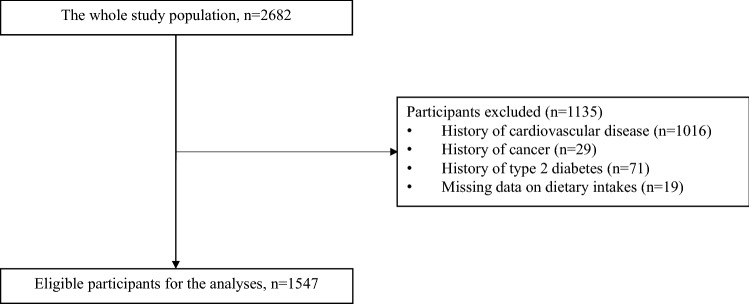
A flowchart showing the reasons for exclusion of participants from the analyses of the current study

The KIHD was designed to investigate risk factors for CVD, atherosclerosis and related outcomes in a prospective, population-based sample of men from eastern Finland [20]. The baseline examinations were carried out in 1984–1989. A total of 2682 men (83% of those eligible) who were 42, 48, 54 or 60 years old at baseline were recruited. The baseline characteristics of the entire study population have been described elsewhere [21]. The KIHD protocol was approved by the Research Ethics Committee of the University of Kuopio. All subjects gave written informed consent for participation. Subjects with history of CVD (*n* = 1016), cancer (*n* = 29) or type 2 diabetes (*n* = 71) at baseline or with missing data on dietary intakes (*n* = 19) were excluded, leaving 1547 men for the current analyses (Fig. 1).

### Assessment of dietary intakes

Consumption of foods at baseline was assessed with an instructed food recording of 4 days, of which one was a weekend day, by household measures. Because the participants recorded their dietary intakes right before their study visit, some participants recorded the intakes over consecutive days, whereas some participants had non-consecutive days of recording. A picture book of common foods and dishes was used to help in estimation of portion sizes. The picture book contained 126 most common foods and drinks consumed in Finland during the 1980s, and for each food item the participant could choose from three to five commonly used portion sizes or describe the portion size in relation to those in the book. To further improve accuracy, instructions were given and completed food records were checked by a nutritionist together with the participant. Nutrient intakes were estimated using the NUTRICA^®^ 2.5 software (Social Insurance Institution, Turku, Finland). The databank of the software is mainly based on Finnish values of nutrient composition of foods.

### Healthy Nordic diet score

The original Baltic Sea Diet Score consists of nine components, of which six are food groups and three represent nutrients [4]. The food items have been selected based on the traditional food culture in the Nordic countries. However, due to the lack of information on certain food items in our database, the contents in the food groups in our study were not identical to those in the original Baltic Sea Diet Score. The original Baltic Sea Diet Score components and those used in the current study are presented in Table 1. The main difference is that for some score components, we used the whole food group instead of individual food items in that group. For example, instead of using rye, oats and barley as the cereal component of the score, we used whole grains (excluding rice and pasta) (Table 1). The score was calculated according to quartiles of consumption for each score component. For the positive score components, the lowest quartile was given 0 points, the second one 1 point, the third one 2 points and the highest quartile of intake 3 points. For the negative score components, points were given in reverse order, except for alcohol, which was given 0–1 point (one point was given if the ethanol intake was < 20 g/day, otherwise zero points were given). The points given for each component were summed up to obtain the overall score. The resulting score ranged from 0 to 25. The higher the score, the healthier was the diet. In the analysis, the score was used both as a continuous variable and categorized into quartiles. The scores < 11 were included in the lowest quartile, scores 11–12 and 13–15 in the two middle quartiles and scores > 15 in the highest quartile.

**Table 1:** The components of the original Baltic Sea Diet Score and those used in the present study and the cutoffs for component intakes

			Healthy Nordic diet score^e^
			Cutoff values in the current study
Score component	Contents of the original Baltic Sea Diet Score [4]	Contents in the current study	
Fruits and berries (g/day)	Berries, apples, pears	All fruits, berries	38; 105; 192
Vegetables (g/day)	Tomato, cucumber, cabbage, roots, peas, lettuce	Roots, pulses, vegetables	63; 105; 161
Cereals (g/day)	Rye, oats, barley	Whole grains^a^	108; 149; 204
Low-fat milk (g/day)	Fat-free milk and milk < 2% fat	Fat-free milk and milk < 2% fat	83; 223; 477
Fish (g/day)	Salmon, freshwater fish	Salmon, freshwater fish	0; 29; 61
Meat products (g/day)	Beef, pork, processed meat products, sausages	Processed and unprocessed meat	88; 130; 185
Total fat (*E*%)^b^	Total fat as a percentage of total energy intake	Total fat as a percentage of total energy intake	35; 39; 42
Fat ratio	Ratio of PUFA^c^ to SFA^d^ + trans-fatty acids	Ratio of PUFA to SFA + trans-fatty acids	0.17; 0.24; 0.31
Alcohol (g/day)^f^	Ethanol	Ethanol	20

^a^Excluding rice and pasta

^b^%, percentage of total energy intake

^c^PUFA, polyunsaturated fatty acids

^d^SFA, saturated fatty acids

^e^The healthy Nordic diet score was calculated using the population-based consumption quartiles as cutoffs, with each intake quartile scored as 0, 1, 2 or 3 points. For the potentially healthy score components (fruits and berries, vegetables, cereals, low-fat milk, fish and fat ratio), the lowest intake category was given 0 points and the highest 3 points. For the potentially less favorable score components (meat products and total fat), the lowest intake category was given 3 points and the highest 0 points

^f^Men consuming 20 g or less of alcohol per day received 1 point; otherwise 0 points were given

### Health examination and measurements

Venous blood samples were collected between 8 and 10 AM at the baseline examinations. Subjects were instructed to abstain from ingesting alcohol for 3 days and from smoking and eating for 12 h prior to giving the sample. Detailed descriptions of the determination of serum lipids and lipoproteins [22], assessment of medical history and use of medications at baseline [22], family history of diseases [22], smoking [22], alcohol intake [22], blood pressure [22] and physical activity [23] have been published. Hypertension was defined as blood pressure over 140/90 mmHg or use of hypertension medication. Body mass index (BMI) was computed as the ratio of weight in kilograms to the square of height in meters. Information on the medication use during the follow-up was obtained from the national Drug Prescription Registry at the Social Insurance Institute. Serum C-reactive protein (CRP) was measured with an immunometric assay (Immulite High Sensitivity CRP Assay, DPC, Los Angeles, CA, USA). Education and income were assessed by self-administered questionnaires.

### Ascertainment of follow-up events

Deaths were ascertained by a computer linkage to the national cause of death register using the Finnish personal identification code (social security number). There were no losses to follow-up. All deaths were coded according to the Tenth International Classification of Disease (ICD) codes. All disease deaths that occurred from the study entry to December 31, 2014 were included. ICD codes I00-I99 and C00-D48 were used to define CVD and cancer deaths, respectively. Deaths due to accidents or suicides (ICD codes S00-T98) during the follow-up were not included, because diet can be considered to have less impact on these outcomes. Among the 1547 men included in these analyses, 62 men died due to accidents or suicides.

### Statistical analysis

Cox proportional hazards regression models adjusted for relevant covariates were used to estimate hazard ratios (HR) of incident events in quartiles of the score. The quartiles were generated automatically in SPSS. Because of the low range of values in the score (2–25), the number of participants in the quartiles did not end up even. The time at risk was from the baseline examinations in 1984–1989 until death or the end of follow-up on Dec 31, 2014. The validity of the proportional hazards assumption was confirmed by using Schoenfeld residuals. The Model 1 included age (years), examination year and energy intake (kcal/day). The multivariable Model 2 included Model 1 and pack-years of smoking, body mass index (kg/m^2^), leisure-time physical activity (kcal/day), education (years), marital status (yes/no) and income (euros/year). All quantitative variables were entered in the models as continuous variables. Cohort mean was used to replace missing values in covariates (2.3% in pack-years of smoking, 1.7% in income, 0–0.5% in others). Tests of linear trend across the quartiles were conducted by assigning the median values for each category of exposure variable and treating those as a single continuous variable*.* Potential nonlinear associations were assessed semiparametrically using restricted cubic splines. All *P* values were two-tailed (*α* = 0.05). Data were analyzed using SPSS 25.0 for Windows (Armonk, NY: IBM Corp.) and Stata 14.1 (Stata Corp., College Station, Texas; for spline analysis).

## Results

The baseline characteristics for the 1547 participants are presented in Table 2. Participants who had higher adherence to the healthy Nordic diet score had higher leisure-time physical activity, education level and lower alcohol intake, serum total cholesterol, LDL cholesterol, triglycerides and CRP concentrations and they were less often smokers and were more likely married compared to the participants with lower adherence to healthy Nordic diet score.

**Table 2:** Characteristics of the 1547 participants according to healthy Nordic diet score

Characteristic	Healthy Nordic diet score			
	1 (2–10)	2 (11–12)	3 (13–15)	4 (16–25)
Number of subjects	432	291	409	415
Age (years)	52.0 (5.3)^a^	52.0 (5.6)	51.8 (5.4)	52.4 (5.5)
Body mass index (kg/m^2^)	26.3 (3.6)	26.2 (3.3)	26.6 (3.4)	26.6 (3.2)
Leisure-time physical activity (kcal/day)	106 (149)	128 (148)	148 (161)	163 (170)
Income (euros)	12,468 (9195)	13,945 (8723)	14,901 (9318)	15,770 (8922)
Education (years)	8.1 (3.0)	8.8 (3.3)	9.3 (3.9)	9.8 (3.9)
Systolic blood pressure (mmHg)	134 (16)	134 (15)	134 (17)	132 (15)
Diastolic blood pressure (mmHg)	89 (10)	89 (10)	89 (11)	88 (10)
Energy intake (kcal/d)	2505 (644)	2535 (693)	2477 (626)	2437 (560)
Alcohol intake (g/week)	89 (130)	77 (107)	73 (123)	52 (94)
Serum total cholesterol (mmol/L)	5.99 (1.06)	5.93 (1.01)	5.81 (0.98)	5.72 (1.05)
Serum LDL cholesterol (mmol/L)	4.12 (1.02)	4.05 (0.98)	3.96 (0.92)	3.90 (0.99)
Serum HDL cholesterol (mmol/L)	1.33 (0.29)	1.34 (0.34)	1.31 (0.28)	1.29 (0.27)
Serum triglycerides (mmol/L)	1.21 (0.77)	1.20 (0.78)	1.21 (0.67)	1.27 (0.74)
Serum C-reactive protein (mg/L)	2.6 (5.9)	2.2 (3.8)	2.1 (3.4)	1.9 (3.9)
Blood glucose (mmol/L)	4.6 (0.5)	4.6 (0.5)	4.6 (0.5)	4.5 (0.5)
Serum insulin (mU/I)	11.0 (6.0)	10.4 (6.0)	10.8 (5.6)	10.3 (5.6)
Hypertension (%)	52.1	55.3	51.6	52.8
Use of hypertension medication during follow-up (%)	74.1	73.9	75.3	75.4
Use of cholesterol medication at baseline (%)	0	0.3	0	0.5
Use of cholesterol medication during follow-up (%)	50.0	48.5	50.1	48.7
Current smoker (%)	45.6	32.0	25.7	19.8
Cancer history in family (%)	22.7	26.5	24.2	26.5
Cardiovascular diseases history in family (%)	77.8	78.0	80.2	79.0
Marital status married (%)	78.5	89.0	89.0	90.6

^a^All values are means (SD) or percentages

**Fig. 2:**
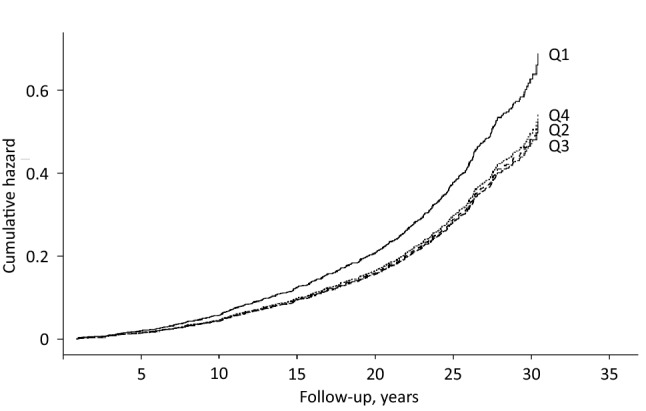
Cumulative hazard of any disease death (*n* = 576) according to the quartiles of the healthy Nordic diet score among 1547 men from the Kuopio Ischaemic Heart Disease Risk Factors Study. The model is adjusted for age (years), examination year, energy intake (kcal/day), pack-years of smoking, body mass index (kg/m^2^), leisure-time physical activity (kcal/day), education (years), marital status (yes/no) and income (euros/year)

**Fig. 3:**
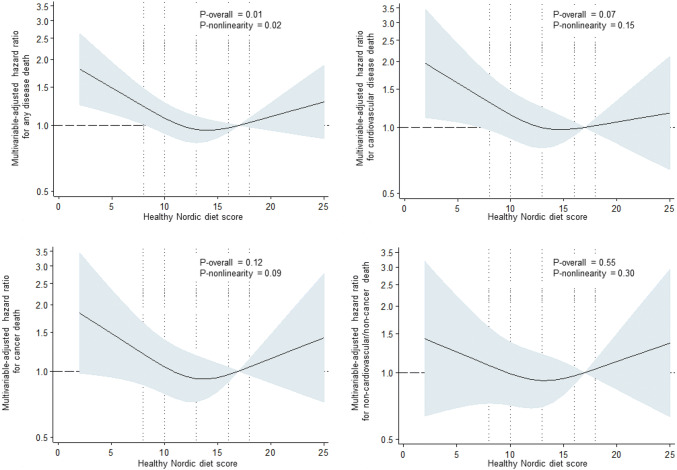
Hazard ratio of the healthy Nordic diet score with risk of disease death among 1547 men, evaluated by restricted cubic splines from Cox proportional hazards models. The model is adjusted for age (years), examination year, energy intake (kcal/day), pack-years of smoking, body mass index (kg/m^2^), leisure-time physical activity (kcal/day), education (years), marital status (yes/no) and income (euros/year). The solid line represents the central risk estimate and the shaded area the 95% confidence interval. The dotted vertical lines correspond to the 10th, 25th, 50th, 75th and 90th percentile of the healthy Nordic diet score

During the average follow-up of 23.6 years (SD 7.0 years), 576 men (37.2%) died due to disease. Of these, 250 (43.4%) were CVD deaths, 194 (33.7%) cancer deaths and 132 (22.9%) other deaths. Of the other deaths, the most common non-CVD/non-cancer causes of death were deaths due to central nervous system diseases such as Alzheimer, Parkinson and dementia (8.3% of all disease deaths), respiratory-related causes (2.6%) and diseases of the digestive system such as liver disease (2.6%). In Model 1 adjusted for age, examination year and energy intake, those in the lowest vs. the highest quartile of the healthy Nordic diet score had 65% (95% CI = 33–106%) higher risk of disease death (*P*-trend across quartiles < 0.001), 77% (95% CI = 27–147%, *P*-trend = 0.001) higher risk of CVD death and 71% (95% CI = 16–153%, *P*-trend = 0.007) higher risk of cancer death (Table 3). There was no statistically significant association with risk of death due to other causes (Table 3). Further adjustment for potential confounders attenuated the associations, but the association with any disease death [27% (95% CI = 1–59%) higher risk in the lowest vs. the highest quartile] and the trend toward higher risk of CVD death (*P*-trend = 0.049) remained statistically significant (Model 2, Table 3 and Fig. 2). However, the restricted cubic splines analysis suggested a possible non-linear association between the healthy Nordic diet and risk of any disease death (Fig. 3). We did not find evidence for non-linearity with the other outcomes (Fig. 3).

**Table 3:** Risk of death in quartiles of the healthy Nordic diet score

	Quartile of the healthy Nordic diet score (range)				*P*-trend
	1 (2–10) (*n* = 432)	2 (11–12) (*n* = 291)	3 (13–15) (*n* = 409)	4 (16–25) (*n* = 415)	
*Disease death*					
Number of events (% of subjects)	201 (46.5)	103 (35.4)	135 (33.0)	137 (33.0)	
Incidence rate/1000 PY	20.67	15.07	13.67	13.53	
Model 1	1.65 (1.33–2.06)	1.12 (0.81–1.31)	1.03 (0.81–1.31)	1	< 0.001
Model 2	1.27 (1.01–1.59)	0.97 (0.75–1.26)	0.95 (0.75–1.21)	1	0.027
*CVD death*					
Number of events (% of subjects)	91 (21.1)	40 (13.7)	60 (14.7)	59 (14.2)	
Incidence rate/1000 PY	9.36	5.85	6.08	5.83	
Model 1	1.77 (1.27–2.47)	1.02 (0.68–1.53)	1.08 (0.75–1.55)	1	0.001
Model 2	1.39 (0.99–1.97)	0.92 (0.61–1.39)	0.99 (0.69–1.42)	1	0.049
*Cancer death*					
Number of events (% of subjects)	68 (15.7)	33 (11.3)	51 (12.5)	42 (10.1)	
Incidence rate/1000 PY	6.99	4.83	5.16	4.15	
Model 1	1.71 (1.16–2.53)	1.14 (0.72–1.80)	1.22 (0.81–1.84)	1	0.007
Model 2	1.26 (0.84–1.89)	0.94 (0.59–1.50)	1.12 (0.74–1.70)	1	0.316
*Other death*					
Number of events (% of subjects)	42 (9.7)	30 (10.3)	24 (5.9)	36 (8.7)	
Incidence rate/1000 PY	4.32	4.39	2.43	3.56	
Model 1	1.38 (0.88–2.18)	1.28 (0.79–2.08)	0.73 (0.43–1.23)	1	0.063
Model 2	1.04 (0.65–1.68)	1.06 (0.64–1.74)	0.67 (0.40–1.13)	1	0.563

Model 1 is adjusted for age (years), examination year and energy intake (kcal/day)

Model 2 is adjusted for Model 1 plus pack-years of smoking, body mass index (kg/m^2^), leisure-time physical activity (kcal/day), education (years), marital status (yes/no) and income (euros/year)

*PY* person-years

In the sensitivity analyses, we included only those men with complete data on all covariates (*n* = 1475). This had little impact on the associations; for example, the HR (95% CI) in the lowest vs. the highest quartile of the healthy Nordic diet score was 1.27 (0.99–1.59, *P*-trend = 0.04) for any disease death (547 events) and 1.37 (0.96–1.96, *P*-trend = 0.07) for CVD death (236 events) (other data not shown).

## Discussion

In this prospective population-based cohort study among middle-aged and older men, the men with the lowest adherence to a healthy Nordic diet had a higher risk of any disease death, possibly attributable to higher CVD mortality. However, there was no evidence for an association with cancer mortality or mortality due to other causes than CVD and cancer, after adjusting for potential confounders.

There are some differences in the diet scores that are used to define a healthy Nordic diet. The original Baltic Sea Diet Score from Finland includes nine commonly used food components in the Nordic countries [4]. They are Nordic fruits, vegetables, cereals, low-fat and fat-free milk, fish, ratio of PUFA to SFA and trans-fatty acids, processed meat, total fat and intake of alcohol (Table 1). Our score was modeled according to the Baltic Sea Diet Score, but included some differences due to lack of information on some individual food items. Another score to define a healthy Nordic diet is the Healthy Nordic Food Index developed in Denmark, which includes rye bread, oatmeal, root vegetables, cabbages, fish, shellfish, apples and pears [11]. The later developed New Nordic Diet Score from Norway has also been used to study the health effects of a healthy Nordic diet. It consists of foods that can be produced in Nordic climate, such as whole grains, root vegetables, cabbages, berries, fruits, fish, potatoes and rapeseed oil [24]. The New Nordic Diet Score also considers the meal frequency and water consumption.

These different versions of a healthy Nordic diet have had beneficial associations with several disease risk factors, such as hypertension [8], abdominal obesity and weight gain [5, 10, 25], inflammation [7] and serum lipids [9], although one study reported an association with decreased HDL cholesterol [26]. Higher adherence to a healthy Nordic diet has also been associated with lower risk of colorectal cancer in women [12], better cognition [27], greater muscle strength and physical capacity in old age [6, 28], and with reduced risk of myocardial infarction [15, 16], stroke [17], total mortality [11, 13, 14] and type 2 diabetes [19], although some studies have observed no association with risk of type 2 diabetes [18] or cardiovascular disease [29]. Overall, however, a higher adherence to a healthy Nordic diet appears to be associated with beneficial health outcomes, and our current findings add to the knowledge of the potential health benefits. A possible explanation for the lack of an association with cancer death or death due to other causes than CVD and cancer is that these outcomes include various types of cancer and causes of death and diet may have only a small impact on some of the outcomes. Unfortunately, the number of events of individual causes of death in these groups was too small for more detailed investigation.

A partial explanation for the findings in our study may also be that the participants who had the lowest adherence to a healthy Nordic diet were also more likely to have lower income and education and more adverse lifestyle factors than participants with higher adherence (Table 2). This is also suggested by the significant attenuation of the associations after controlling for several of these factors, so that only the association with any disease death and the trend toward lower CVD death risk remained statistically significant. However, previous studies in the KIHD cohort have shown an association between several individual components of a healthy Nordic diet and mortality risk. In the KIHD cohort, higher meat consumption has been associated with increased risk of death [30] and higher intake of fruits and berries with reduced risk [31]. In addition, higher PUFA intake has been associated with a lower risk of coronary heart disease death, whereas total fat intake was not associated with the risk [32]. Intake of milk or fish have not been associated with mortality risk [30]. Thus, a score may include components that do not have an association with disease risk in a certain study population, which then attenuates the association with the composite score.

The strengths of our study include the population-based cohort setting, comprehensive information about viable confounding factors and no loss to follow-up. Our study also has limitations. A major limitation is that the dietary intakes were assessed only once at the baseline in 1984–1989, which may not accurately reflect the dietary intakes during the long follow-up. For example, of the food components in our healthy Nordic diet score, milk intake has significantly decreased and intakes of poultry and fruits, berries and vegetables have increased during the KIHD follow-up [33, 34]. Also, the total and saturated fat intakes have decreased [34]. Furthermore, even though food recording is regarded as the golden standard for dietary assessment, it has limitations with food items that are only occasionally consumed. For example, the typical intakes of fish and processed meat might have not been captured in our study with 4-day food recording. Finally, because the subjects filled only one food record, we do not have information about the repeatability of the record as a measure of dietary intakes in this study population. These limitations in dietary assessment are a source of random error, which would attenuate the true associations between dietary factors assessed at baseline and risk of an event during the follow-up. Because the outcome was not all-cause mortality, it cannot be assumed that the risk of death equals the rate of death, because of competing events. Finally, because our study population included only men, the results of this study cannot necessarily be generalized to women.

In conclusion, among middle-aged and older men in eastern Finland, low adherence to a healthy Nordic diet was associated with a higher risk of disease death, which is possibly explained by higher CVD mortality. Because of the limited research data, more studies are needed to investigate the impact of a healthy Nordic diet on disease outcomes.
